# Identification, functional characterization and developmental regulation of sesquiterpene synthases from sunflower capitate glandular trichomes

**DOI:** 10.1186/1471-2229-9-86

**Published:** 2009-07-06

**Authors:** Jens C Göpfert, Gillian MacNevin, Dae-Kyun Ro, Otmar Spring

**Affiliations:** 1 University of Hohenheim, Institute of Botany (210), Garbenstrasse 30, 70599 Stuttgart, Germany; 2 Department of Biological Sciences, University of Calgary, Calgary, T2N 1N4, Canada

## Abstract

**Background:**

Sesquiterpene lactones are characteristic metabolites of Asteraceae (or Compositae) which often display potent bioactivities and are sequestered in specialized organs such as laticifers, resin ducts, and trichomes. For characterization of sunflower sesquiterpene synthases we employed a simple method to isolate pure trichomes from anther appendages which facilitated the identification of these genes and investigation of their enzymatic functions and expression patterns during trichome development.

**Results:**

Glandular trichomes of sunflower (*Helianthus annuus *L.) were isolated, and their RNA was extracted to investigate the initial steps of sesquiterpene lactone biosynthesis. Reverse transcription-PCR experiments led to the identification of three sesquiterpene synthases. By combination of *in vitro *and *in vivo *characterization of sesquiterpene synthase gene products in *Escherichia coli *and *Saccharomyces cerevisiae*, respectively, two enzymes were identified as germacrene A synthases, the key enzymes of sesquiterpene lactone biosynthesis. Due to the very low *in vitro *activity, the third enzyme was expressed *in vivo *in yeast as a thioredoxin-fusion protein for functional characterization. In *in vivo *assays, it was identified as a multiproduct enzyme with the volatile sesquiterpene hydrocarbon δ-cadinene as one of the two main products with α-muuorlene, β-caryophyllene, α-humulene and α-copaene as minor products. The second main compound remained unidentified. For expression studies, glandular trichomes from the anther appendages of sunflower florets were isolated in particular developmental stages from the pre- to the post-secretory phase. All three sesquiterpene synthases were solely upregulated during the biosynthetically active stages of the trichomes. Expression in different aerial plant parts coincided with occurrence and maturity of trichomes. Young roots with root hairs showed expression of the sesquiterpene synthase genes as well.

**Conclusion:**

This study functionally identified sesquiterpene synthase genes predominantly expressed in sunflower trichomes. Evidence for the transcriptional regulation of sesquiterpene synthase genes in trichome cells suggest a potential use for these specialized cells for the identification of further genes involved in the biosynthesis, transport, and regulation of sesquiterpene lactones.

## Background

Sesquiterpenoids, widely distributed in the plant kingdom, are 15 carbon natural products derived from farnesyl diphosphate (FDP) by the reactions of sesquiterpene synthases via carbocation intermediates [1-3]. Sesquiterpene hydrocarbons are volatile compounds and well known for their contribution to the scent of essential oils or ripe fruits [4,5]. Many of these compounds show cyclic chemical structures and exhibit a wide range of biological properties from herbivore defence to signalling info-chemicals in plant-insect interactions [6-12]. Biosynthesis of these compounds occurs in various plant organs like conifer resin ducts, leaves, roots, or fruits [13-16].

Sesquiterpene lactones (STLs) are a sub-class of sesquiterpenoids and are typical secondary compounds of the Asteraceae plant family [17,18]. Because of their diverse cytotoxic properties, these compounds are generally separated from the cellular metabolism and stored in specialized cells and compartments like laticifers or glandular trichomes [19,20]. STLs show anti-microbial activities and, due to their bitter taste, serve as anti-feedants [21-23]. From sunflower, *Helianthus annuus*, several STLs with germacranolide skeleton have been identified from capitate glandular trichomes, which are found on various aerial plant parts, particularly on leaves and anthers of the disk florets [24,25].

In the last several years, the potent bioactivities of STLs, such as anti-malarial activity of artemisinin [26], drew significant interests in the biochemistry of STLs. The first committed step in biosynthesis of all cyclic sesquiterpenes and STL is the cyclization of FDP by sesquiterpene synthases in the cytosol [24,25,27,28]. Germacrene A synthases, catalyzing the first step of STL biosynthesis [29], have been identified from different Asteraceae species in the young leaves of *Ixeris dentata *[30], etiolated chicory heads [31], or whole lettuce seedlings [32]. From a glandular trichome cDNA library of *Artemisia annua*, a germacrene A synthase has been identified which is the first and so far only sesquiterpene synthase which was isolated directly from secretory glandular cells [33].

On the leaves of *Helianthus annuus*, two different types of glandular trichomes are observed: uniserial glandular trichomes producing sesquiterpenes with bisabolene skeleton [34] and capitate glandular trichomes which secrete STL of the germacranolide type [35] and 5-deoxy-flavons [36] into an apical cuticular globe. Generally, trichomes on the leaves of Asteraceae plants develop in early leaf stages. This strongly complicates the isolation of these specialized chemical factories for identification of enzymes involved in sesquiterpene biosynthesis and does not allow a stage-specific analysis of gene expression and secretion process. Besides the sunflower leaves, the same glandular trichomes are found at the anther tips of disk florets [37]. Trichome development in these flower parts proceeds simultaneously with the floret and pollen growth, thus allowing an exact visual assessment of the trichome developmental stages [38]. It was shown by microscopic observations and HPLC analysis that secretion of STL takes place during the expansion of the subcuticular globe. Using a micropreparation technique, sunflower capitate glandular trichomes from anthers could be isolated in specific developmental stages ranging from the pre- to the post-secretory phase. The accessibility of differentially developed trichomes by means of this simple and effective micropreparation technique enabled us to use the sunflower inflorescence as a promising model system to study sesquiterpene lactone metabolism.

Besides the germacrene A synthases, only a limited number of terpene synthases have been described from Asteraceae. Two enantiospecific (+)- and (-)-germacrene D synthases from *Solidago canadensis *[39] have been characterized. In addition, caryophyllene-, epi-cedrol- and amorpha-4,11-diene synthases were isolated from young leaves of *Artemisia annua *[40-42].

In this paper, we report the isolation of three sunflower sesquiterpene synthases and their functional characterization *in vitro *after expression in *Escherichia coli *and *in vivo *in *Saccharomyces cerevisiae*. Furthermore their expression patterns in different sunflower tissues and in different biosynthetic stages during trichome development is analyzed.

## Results and discussion

### Identification of sunflower sesquiterpene synthases

Sunflower capitate glandular trichomes were isolated in the biosynthetically active stage from anther appendages as previously described [36]. Degenerated primers for the conserved sequences of sesquiterpene synthases [43] were used in reverse transcription (RT)-PCR to retrieve partial sesquiterpene synthase sequences. The deduced amino acid sequences of the two PCR fragments displayed high sequence similarity to other sesquiterpene synthase genes in the public database. The 3'- and 5'-rapid amplification of cDNA ends (RACE) led to the identification of two distinct full-length cDNAs. The mRNA for *HaTPS1 *[GenBank: DQ016667] showed a length of 1,913 bp, containing an ORF of 1,680 bp coding for 559 amino acids. The mRNA sequences for *HaTPS2 *[GenBank: DQ016668] showed a length of 1,944 bp with an ORF of 1,668 bp coding for 555 amino acids. Both genes encoded proteins with high similarity to germacrene A synthases from other plant species. The molecular weight of the enzymes was calculated to 64.4-kDa for *HaTPS1 *and 64.2-kDa for *HaTPS2*, falling into the typical mass range of plant sesquiterpene synthases [43-45].

**Figure 1:**
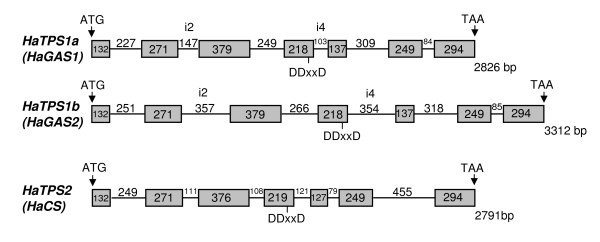
**Display of the exon-intron structure of *HaTPS1a *(*HaGAS1*), *HaTPS1b *(*HaGAS2*) and *HaTPS2 *(*HaCS*) genes**. Grey boxes represent exon sequences, lines show intron sections. Length of nucleotide sequences is shown for exons in the boxes and for introns above the line. DDxxD marks the position of the conserved aspartate-rich region. i2: intron 2; i4: intron 4.

**Figure 2:**
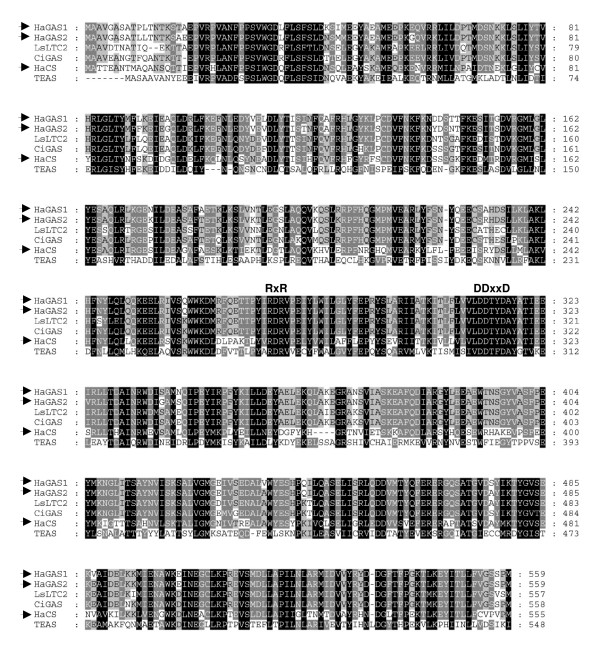
**Alignment of the deduced amino acid sequences of sesquiterpene synthase genes**. *HaTPS1a (HaGAS1), HaTPS1b (HaGAS2)*, and *HaTPS2 (HaCS) *indicate sesquiterpene synthase genes isolated from sunflower in this study. *LsLTC2*, *CiGAS*, and *TEAS *are germacrene A synthase from *Lactuca sativa*, germacrene A synthase from *Cichorium intybus*, and 5-epi-aristolochene synthase from *Nicotiana tabacum*, respectively. Black: identical amino acids in all sequences; dark grey and light grey: identical amino acids in 5 or 4 enzymes, respectively.

For further systematic classification of the identified sesquiterpene synthases, the genomic DNA (gDNA) sequences were obtained. PCR amplification of gDNA with specific primers for *HaTPS2 *resulted in a single amplicon. The complete nucleotide sequence showed a length of 2,791 bp [GenBank: EU443250]. In contrast to *HaTPS2*, PCR amplification with primers for *HaTPS1 *showed two gDNA fragments which differed in length. Sequencing of both products resulted in the identification of a third sesquiterpene synthase that showed 95% deduced amino acid identity to the *HaTPS1*. Thus, the ORF of the *HaTPS1 *and its close homolog was designated as *HaTPS1a *and *HaTPS1b *[GenBank: EU327785], respectively. The gDNA sequences for *HaTPS1a/b *consisted of highly similar exon structures but differed considerably in their intron length and sequence in particular for the intron 2 and 4 (Figure 1). The gDNA for *HaTPS1a *showed a length of 2,826 bp [GenBank: EU439590] while the gDNA for *HaTPS1b *contained 3,312 bp [GenBank: EU443249]. All three sunflower sesquiterpene synthase genes displayed an assembly of 7 exons and 6 introns, typical for class III terpene synthases [46]. They belong to the *TPSa*-gene family [45], a group of genes mainly consisting of angiosperm sesquiterpene synthases [47]. The ORF size in *HaTPS1b *was identical to *HaTPS1a *(559 amino acids) with 95% sequence identity. The *HaTPS1a/b *shared 63% amino acid identity with *HaTPS2 *and shared 94% similarity to germacrene A synthases from Asteraceae like *Lactuca sativa *(*LsLTC1 *and *LsLTC2*; [32]) or *Cichorium intybus *(*CiGASl*o [31]). Comparison of the deduced amino acid sequence of *HaTPS2 *with entries in public database showed 62% similarity to Asteraceae germacrene A synthases from lettuce or chicory but showed only about 40% identity to δ-cadinene synthases from *Gossypium arboretum *and *G. hirsutum *[48-50]. The amino acid sequences of all three sesquiterpene synthases contained the common sequence motifs for the sesquiterpene synthase family [45], such as the highly conserved DDxxD and RxR motifs, responsible for the divalent metal ion-substrate binding (Figure 2).

### Functional characterization of sunflower sesquiterpene synthases *HaTPS2 (HaCS)*

**Figure 3:**
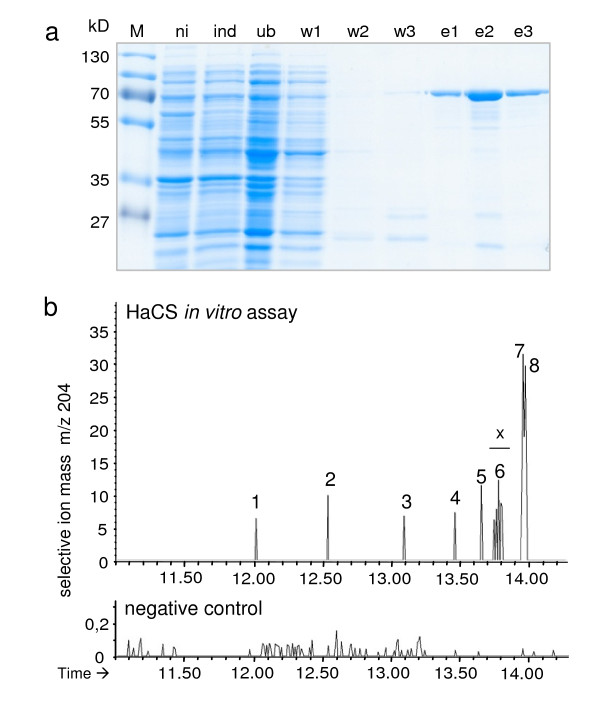
**(a) PAGE showing purification of recombinant HaCS-fusion protein using Ni-NTA affinity chromatography**. ni: uninduced control; ind: induced *E. coli *culture; ub: unbound fraction; w1-2: washing steps 1 to 2 using 20 mM imidazol; w3: washing step 3 using 100 mM imidazol; e1-3: elution steps 1 to 3 using 250 mM imidazol; M: marker. (b) GC-MS analysis (m/z 204) of an *in vitro *incubation of recombinant HaCS protein with FDP. Negative control: incubation of an *E. coli *protein extract not expressing HaCS under the same conditions. x: unidentified compounds.

**Figure 4:**
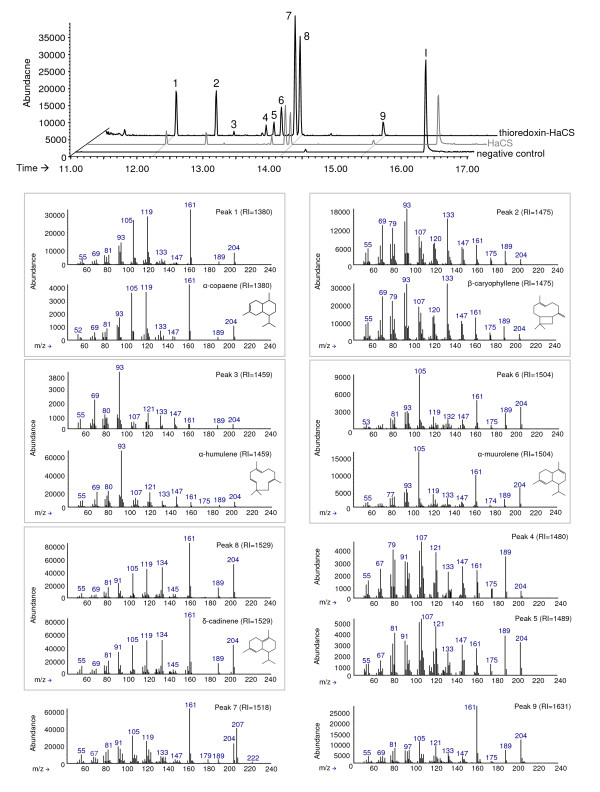
**GC-MS analysis of sesquiterpene products of the *in vivo *expression of *HaCS *in *S. cerevisiae***. Diagrams show products obtained by the expression of *HaCS *and thioredoxin-*HaCS *in comparison to pESC-Leu2d empty vector (negative control). Peak numbers in each panel correspond to numbers above peaks in the chromatogram. Mass spectra of identified products are shown in the top of each panel with a mass spectrum of an authentic standard below. One of the two main products was identified as δ-cadinene; the fragmentation pattern of peak 7 showed high similarities to γ-cadinene but differed slightly in the retention time. RI: retention indice. I: farnesol.

For functional characterization, all three synthase genes were heterologously expressed in *E. coli *as thioredoxin-fusion proteins. The *HaTPS2 *fusion protein was purified through Ni-NTA affinity chromatography (Figure 3a). The gas chromatograph mass spectroscopy (GC-MS) analysis of the *in vitro *enzyme assay products with substrate FDP showed the presence of the parental mass of sesquiterpene (m/z 204) but with a very low product yield (Figure 3b) which prevented the identification of conversion products. To solve this problem, *HaTPS2 *was expressed heterologously *in vivo *in *S. cerevisiae *using high-copy plasmid pESC-Leu2d in the EPY300 strain which was previously engineered to synthesize copious amount of FDP from simple carbon source [51,52]. The transgenes were induced by the addition of galactose to the medium, and non-volatile dodecane was overlaid on the culture to trap volatile sesquiterpene products. The GC-MS analysis of recombinant enzyme product showed a much higher product yield compared to the *in vitro *assay (Figure 3), but the fragmentation pattern of the obtained products still did not allow unambiguous product identification. In this analysis, significant accumulation of farnesol was detected in the negative control (Figure 4, peak I) and in the products obtained by expression of *HaTPS2 *in EPY300, indicating an incomplete conversion of FDP to sesquiterpenes. To improve the activity of the enzyme, *HaTPS2 *was expressed as a thioredoxin-fusion protein (thioredoxin-HaTPS2, Figure 4) in EPY300, similar to the expression in *E. coli*. This modification resulted in a notable increase of synthesized products without altering the overall product profile. Farnesol was completely absent in this *in vivo *assay, indicating a complete conversion of FDP to sesquiterpenes probably due to the significantly enhanced *HaTPS2 *activity. Products of *HaTPS2 *were identified by comparison with the reference spectra in the NIST02 library and also with the authentic standards.

One of the two main products was identified as δ-cadinene with α-muurolene, β-caryophyllene, α-humulene and α-copaene as minor products. This result characterized *HaTPS2 *as an Asteraceae sesquiterpene synthase involved in the biosynthesis of mainly δ-cadinene and other minor sesquiterpenes (Figure 4), and hence it was named as *HaCS *(*Helianthus annuus *Cadinene Synthase). The second main product remained unidentified, but its fragmentation pattern (Figure 4, peak 7) showed high similarities to that of an authentic γ-cadinene standard, but differed slightly in the retention time indices (RIs). The RI of authentic γ-cadinene was 1519, whereas the RI of peak 7 was 1521. Spiking the yeast terpene products with the standard showed a fused, broader peak than the original peak 7 or γ-cadinene standard, indicating that peak 7 is not γ-cadinene (data not shown). Comparison of this fragmentation pattern with that of δ-cadinene and γ-cadinene indicates that this so far unidentified compound is also a product with a cadinene skeleton, but its chemical identity remains unknown. The fragmentation patterns of peaks 4 and 5 were very similar to each other (Figure 4) and both showed high similarities to the fragmentation pattern of γ-gurjunene from the NIST02 library.

Multiproduct terpene synthases are known from different plant families. For example, γ-humulene synthase and δ-selinene synthase from *Abies grandies *produce 52 and 34 products, respectively, from the FDP [53]. Within Asteraceae, only the epi-cedrol synthase from *Artemisia annua *[42] has been described to produce more than one product in significant amount. The only other two sesquiterpene synthases producing sesquiterpenes with the cadinene skeleton were found in *Gossypium hirsutum *and *G. arboretum *[48,49,54,55].

GC-MS analysis of pentane extracts from isolated glandular trichomes showed small amounts of sesquiterpenes. Peaks representing α-copaene, β-caryophyllene, δ-cadinene and the unidentified compounds 7 and 9 (Figure 4) were detected. SPME headspace-analysis of sunflower volatiles showed a whole bouquet of different mono- and sesquiterpenes. Again sesquiterpenes produced by *HaCS *were detected, this time in larger amounts (data not shown). These results are in accordance with the report from Schuh et al. [56] where β-caryophyllene and α-humulene were detected as volatile emissions from *H. annuus*. Besides the compounds produced from *HaCS*, several other sesquiterpenes were detected in the headspace experiments, indicating the activity of yet unidentified terpene synthases in sunflower. Generally, terpene synthases form a large gene family [47,57], and hence it is evident that more terpene synthases are expressed in different sunflower tissues or still remained unidentified in glandular trichomes.

### Functional identification of *HaTPS1a *and *HaTPS1b *as germacrene A synthases (*HaGAS*)

**Figure 5:**
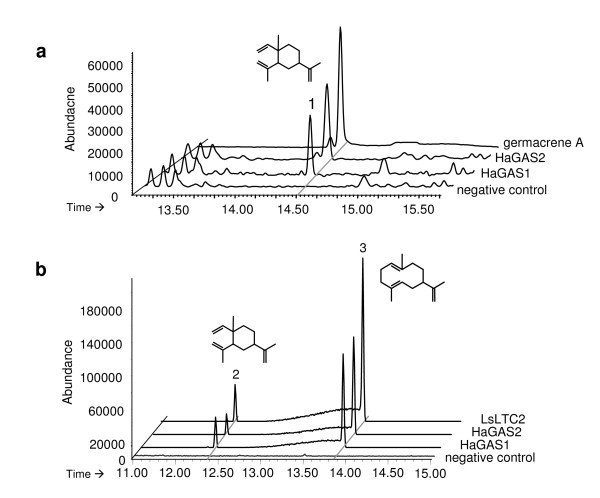
**GC-MS analysis of sesquiterpene products of sunflower germacrene A synthases HaGAS1 and HaGAS2 compared to germacrene A standard produced by LsLTC2 from lettuce**. (a) *In vitro *enzyme-substrate reactions using purified recombinant HaGAS1 or HaGAS2 with the substrate FDP showing β-elemene (peak 1), the cope-rearrangement product of germacrene A. Germacrene A: standard produced by expression of *LsLTC2 *(*L. sativa *germacrene A synthase 2, chromatogram not true to scale); negative control: incubation of FDP with total proteins isolated from *E. coli *without plasmid. (b) *In vivo *products from gene expression in yeast showing β-elemene (peak 2) and germacrene A (peak 3). Negative control: yeast strain carrying the empty vector. Differences between (a) and (b) were caused by the use of different injection port temperatures.

*HaTPS1a *and *HaTPS1b *were expressed as thioredoxin fusion proteins in *E. coli *to produce soluble protein. *In vitro *enzyme assays with purified enzyme showed a single product with the expected mass of m/z 204 in the GC-MS measurements (Figure 5a). A peak with the same retention time and fragmentation pattern was observed by the analysis of a germacrene A standard, produced by the expression of the previously characterized germacrene A synthase *LsLTC2 *from *Lactuca sativa *[32]. The identity of the peak from the *in vitro *assays was determined as β-elemene by an authentic standard. β-elemene is the known cope-rearrangement product of germacrene A due to the hot injection port temperature for GC measurements [29,32]. Thus, the *HaTPS1a *and *HaTPS1b *were named as *HaGAS1 *and *HaGAS2*, respectively.

The performance of both sunflower germacrene A synthases was tested *in vivo *by expression in yeast and for comparison of the product spectra between *in vitro *and *in vivo *expression. *HaGAS1 *and *HaGAS2 *were cloned into the pESC-Leu2d plasmid and used to transform *S. cerevisiae *EPY300. To produce the germacrene A reference standard, a previously characterized germacrene A synthase from *Lactuca sativa *(*LsLTC2 *[32]) was also cloned and expressed under the same conditions. GC-MS analyses of these products showed two identical peaks for all three germacrene A synthases (Figure 5b). The earlier eluting peak was identified as β-elemene, whereas the later peak represented germacrene A, as it showed identical fragmentation pattern to the literature data and terpene products from reference gene (i.e. *LsLTC2*) [29,31,32]. In contrast to the GC-MS measurements of the *in vitro *assays, germacrene A produced from *in vivo *assays was not completely converted to β-elemene. The complete rearrangement of germacrene A to β-elemene depicted in Figure 5a is likely due to the high injection port temperature used (280°C for Figure 5a versus 250°C for Figure 5b). These results showed that *HaGAS1 *and *HaGAS2 *are germacrene A synthases, which catalyze the first committed reaction for sesquiterpene lactone biosynthesis in sunflower. In contrast to *HaCS*, both germacrene A synthases are single product enzymes.

### Biochemical characterization of sunflower germacrene A synthases

To obtain native HaGAS1 and HaGAS2 enzymes, the *E. coli*-expressed fusion proteins were digested with enterokinase and further purified to remove the thioredoxin and the enterokinase before biochemical characterization. SDS-PAGE showed nearly homogeneous proteins. For determination of the influence of the thioredoxin fusion part on the biochemical properties of HaGAS1, thioredoxin-HaGAS1 fusion protein was also characterized. HaCS did not show reproducible results *in vitro *and its biochemical properties were not determined.

Measured pH-optima were 7.4 for thioredoxin-HaGAS1, 7.7 for native HaGAS1 and 7.5 for native HaGAS2. Apparent *K*_m _values for FPP were calculated as 0.82 μM for HaGAS1, 1.06 μM for thioredoxin-HaGAS1 and 0.74 μM for HaGAS2, indicating a similar range like previously reported for sesquiterpene synthases [58,59]. These results indicate that the fused thioredoxin protein part has only minor effects on the properties and catalytic activity of HaGAS1. In contrast to thioredoxin-HaGAS1 and native HaGAS1, HaGAS2 showed substrate inhibition characteristics; because the activity decreased with substrate concentrations above 2.5 μM. Substrate inhibition has also been reported for monoterpene synthases from *Citrus limon *[60].

### Tissue-specific expression of sunflower sesquiterpene synthases

**Figure 6:**
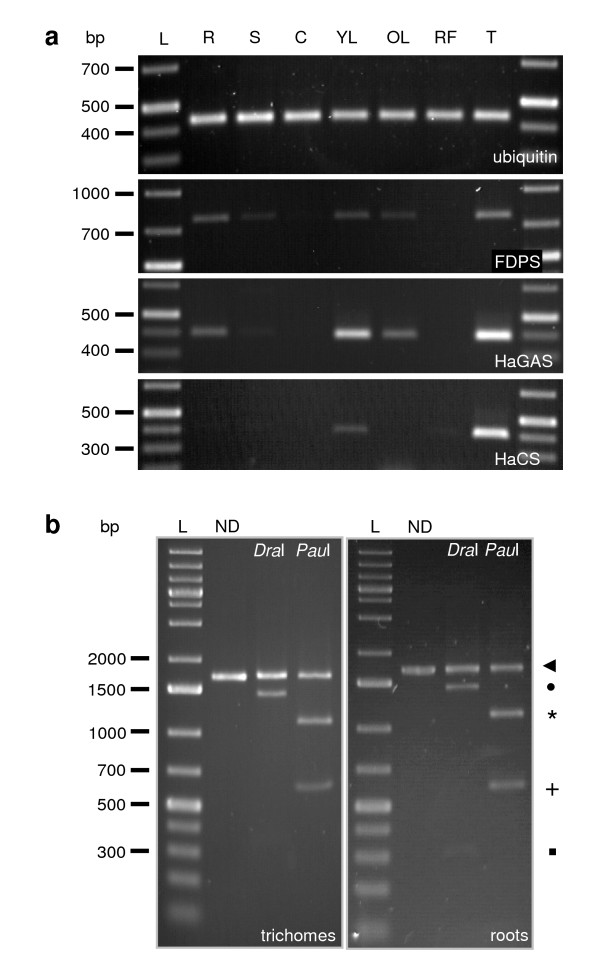
**Expression of sesquiterpene synthase genes in different sunflower tissues**. (a) RT-PCR analyses of germacrene A synthases, *HaCS*, farnesyl diphosphate synthase, and ubiquitin gene expression. Total RNA was extracted from roots (R), stems (S), cotyledons (C), young leaves (YL), old leaves (OL), ray flowers (RF), and capitate glandular trichomes (T). The constitutively expressed gene for ubiquitin was used as cDNA loading control and internal standard. Due to high sequence similarity differentiation between *HaGAS1 *and *HaGAS2 *was not possible by PCR. (b) Detection of the expressed genes for *HaGAS1 *and *HaGAS2 *in trichomes (left) and roots (right) by selective restriction digestion of full length *HaGAS1/2 *cDNA (◂). *HaGAS1 *contains a *Pau*I but no *Dra*I recognition site while *HaGAS2 *contains a *Dra*I site but no *Pau*I recognition site.*Dra*I specifically cuts the amplicon of *HaGAS1 *(1680 bp) into a 1406 bp (•) and 274 bp (▪) fragment. *Pau*I specifically cuts the 1680 bp *HaGAS2 *amplicon into 1105 (*) and 575 bp (+) fragments. ND: undigested control. L: marker.

**Figure 7:**
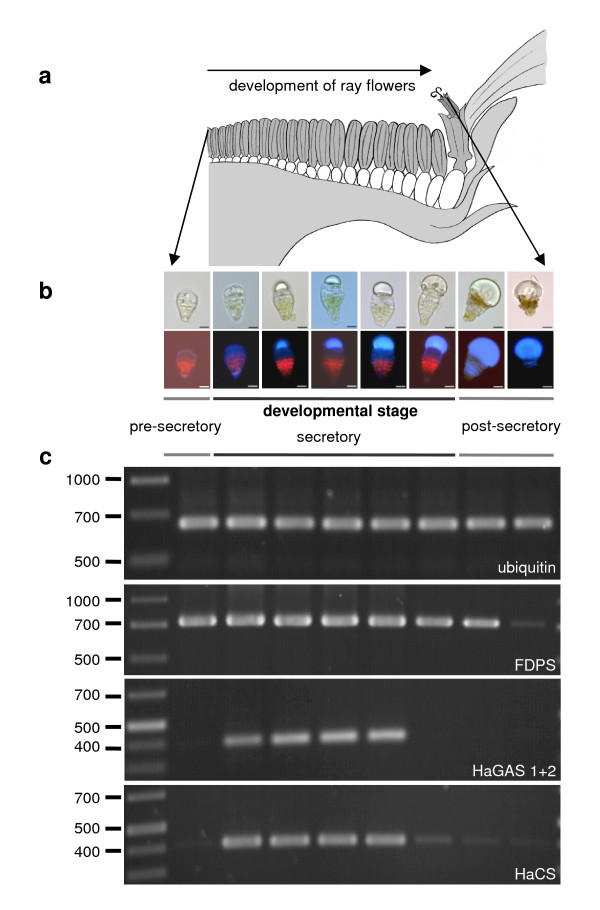
**RT-PCR analysis of *HaGAS*, *HaCS*, and *FDPS *gene expression in different developmental trichome stages**. **(a) **Cross section of a sunflower capitulum showing young florets in the centre and older florets at the margin of the capitulum. **(b) **Micrographs of florets in differently developed trichome stages, as found in the capitulum. **(c) **Semi-quantitative RT-PCR experiments for identification of secretory active trichome stages. Ubiquitin was used as internal standard and loading control; *FDPS*: cDNA amplification of the expressed farnesyl diphosphate synthase gene; *HaGAS*: amplicons for *H. annuus *germacrene A synthases. *HaCS*: amplicons for *H. annuus *cadinene synthase.

**Figure 8:**
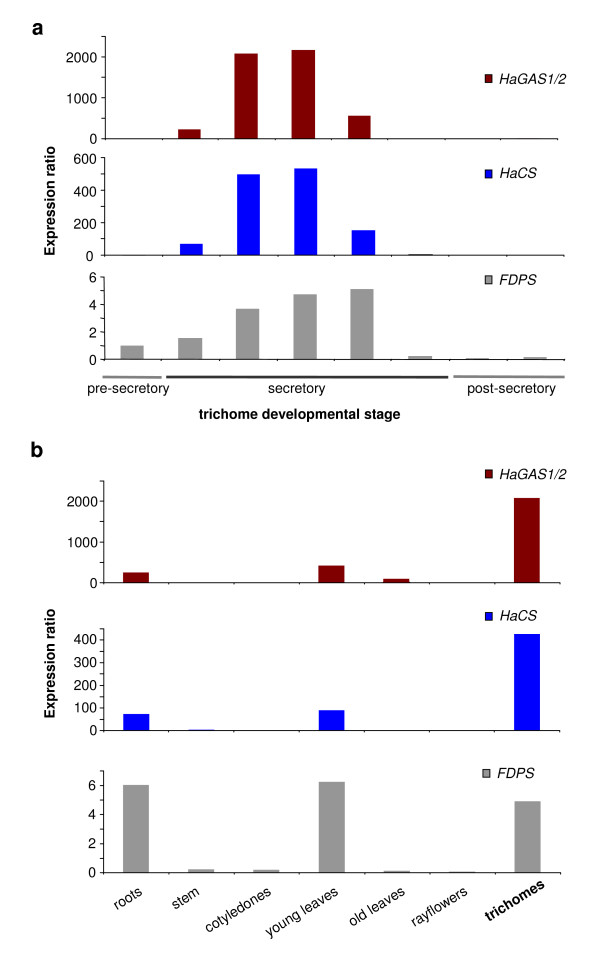
**Quantitative real-time RT-PCR analysis**. **(a) **qPCR data for expression levels of HaGAS1/2, HaCS and FPPS in differently developed trichomes. Same samples were used for generation of these data and those of figure 7. The expression level is shown in comparison to the presecretory trichome stage. **(b) **qPCR analysis of the expression level of the indicated genes in different sunflower tissue. Expression level in presecretory trichome stage was used as reference.

In order to assess the relative transcript level of sesquiterpene synthase, semi-quantitative RT-PCR experiments were carried out using cDNA templates from roots, stem, cotyledons, young and old leaves, ray flowers, and trichomes (Figure 6a). The transcript level for farnesyl diphosphate synthase (*FDPS*) was also analyzed as this enzyme provides the substrate farnesyl diphosphate (FDP) for the sesquiterpene synthases. It could be shown that *FDPS *was expressed in all tissues observed, but was upregulated in those tissues where expression of sesquiterpene synthase genes was detected at highest level (Figures 6, 7, 8). Due to very high sequence similarity, the transcripts for *HaGAS1 *and *HaGAS2 *could not be distinguished in the RT-PCR analyses. Therefore, PCR products for *HaGAS *represent the transcripts from both germacrene A synthase genes. The *FDPS *was detectable with different expression intensity in all organs except for cotyledons. *HaGAS *and the *HaCS *showed a much more differentiated expression. Transcripts of all three genes were traceable in roots and young leaves, but in contrast to *HaGAS*, *HaCS *was not expressed in old leaves. The strongest expression of all three genes was detected in capitate glandular trichomes. These results showed the predominant expression of sunflower sesquiterpene synthase genes in the highly specialized trichome stalk cells. As the transcript for *FDPS *was also detectable in RNA samples from these secretory cells, it is predicted that the whole biosynthetic pathway from the precursor molecules to the secreted STL is located in the sunflower trichomes. Transcripts detected in the leaf samples are likely to show the expression of the sesquiterpene synthase genes in trichomes located on the leaf epidermis. The expression of the sesquiterpene synthase genes in young roots, even though low in comparison to the expression in trichomes, showed that both *HaGAS1/2 *and *HaCS *were not exclusively expressed in the aerial parts of the sunflower. These results were confirmed by quantitative real-time RT-PCR experiments (Figure 8). Expression of terpene synthases in roots has been described for many plant species and seems to be a common biosynthetic process in plants [57,58,61-63].

As the *HaGAS1 *and *HaGAS2 *were not distinguishable by PCR, a restriction digestion was performed to confirm the expression of both genes. The full-length cDNA of both synthases was amplified from root and trichome cDNA with the same primer pair, and their amplicons were subjected to the differential restriction enzyme digestion. The restriction enzyme *DraI *was identified to cut only *HaGAS1 *but not *HaGAS2*, *PauI *cut HaGAS2 but not *HaGAS*. Gel separation of the digested products clearly demonstrated the expression of both germacrene A synthases in roots and trichomes (Figure 6b).

Two lettuce germacrene A synthases were induced in cotyledons by infection with the downy mildew pathogen *Bremia lactucae *[32]. In contrast, inoculation of sunflower seedlings with *Plasmopara halstedii *[64], the sunflower downy mildew pathogen, did not induce the expression of sunflower sesquiterpene synthases in cotyledons or stem and did not have influence on the expression intensity in roots (data not shown). This indicates that expression of these genes is tightly regulated by the developmental program of the trichome secretory cells and is not induced by the infection with these pathogens.

### Developmentally regulated expression of sesquiterpene synthase genes in sunflower capitate glandular trichomes

The low density of glandular trichomes and the early termination of the secretory active phase in young leaf stages complicate the isolation of biosynthetically active trichomes from sunflower leaves. However, trichomes from the anther appendages provided an excellent alternative to study developmental gene expression along the trichome development [38]. Using glands from disk florets of different ages, trichome RNA could be isolated from the pre-secretory to post-secretory stage as previously described [36]. In order to assess transcriptionally active stages of sunflower trichome cells, semi-quantitative RT-PCR experiments were performed to amplify *HaGAS*, *HaCS*, and *FDPS *(Figure 7). While *FDPS *was expressed with the same level in all but the last trichome stage, *HaGAS *and *HaCS *were highly regulated and expressed only during the active secretory phase of the capitate glandular trichomes. Real-Time RT-PCR data indicated that the combined expression level of *HaGAS1 *and *HaGAS2 *seems to be significant higher than that of *HaCS *(Figure 8). A stage-specific expression of terpene synthase genes during trichome development has not been reported before. These semi-quantitative RT-PCR results are in line with the biosynthetically active phases of sunflower trichomes determined by microscopic observations of fluorescent flavones in trichomes and HPLC-measurements of STL content [36,38].

## Conclusion

Three sesquiterpene synthase genes isolated from pure trichomes were functionally expressed in *E. coli *and *S. cerevisiae*. The terpene product analysis by GC-MS showed that they encode two distinct types of sesquiterpene synthases – germacrene A synthase (*HaGAS1/2*) and δ-cadinene synthase (*HaCS*). The expression of *HaGAS1/2 *and *HaCS *in engineered yeast significantly increased the yield of terpene production and thus benefited the identification of terpenes without the need to purify enzymes and to use expensive substrate. All three sesquiterpene synthases were predominantly expressed in the stalk cells of capitate glandular trichomes at active secretory stages. The use of trichomes from anther appendages provides an excellent model system for the analysis of differentially expressed genes during STL biosynthesis. Since the trichomes can be isolated at specific biosynthetic stages, the isolated trichomes can be used to generate expressed-sequence tags or microarray probes, hence providing valuable experimental tools to study STL biochemistry in Asteraceae.

## Methods

### Plant materials, RNA isolation, and cDNA synthesis

*Helianthus annuus *L. cv. HA300 and *Lactuca sativa *var. *capitata *plants were grown under greenhouse conditions with an additional 16 h illumination (330 μmol s^-1 ^m^-2^) and a night length of 8 hours. *H. annuus *capitate glandular trichomes were mechanically isolated from anther appendages as previously described [38]. Studies on the organ development revealed that the formation of trichomes on anthers starts early and parallels the consecutive centripetal maturation of florets within the capitulum. Different trichome stages were determined microscopically by direct analysis of trichomes and pollen development; and light microscopic images were taken as previously described [38].

For total RNA extraction, trichomes were isolates from fresh plant material and immediately transferred to 200 μl ice-chilled RNA extraction buffer (Aurum total RNA isolation Kit, Biorad, Munich, Germany) in 2 ml vials. A mixer mill (MM20, Retsch, Haan, Germany) was used for cell disruption (16 Hz, 1 min) using 2 ceramic beads (2.8 mm diameter, Precellys, Peqlab, Erlangen, Germany). After cell disruption, an additional 500 μl lysis buffer was added. All subsequent steps were carried out as described in the manual. For total RNA isolation from pure glands, glandular trichomes from 200 florets (approx. 30,000 to 40,000 trichomes) were used. For the purification of RNA from different trichome stages, the glands from anther appendages of 50 florets were isolated. RNA quantity and integrity was verified by the Bioanalyzer 2100 using a RNA 6000 Pico Chip (Agilent, Böblingen, Germany). For routine PCR, cDNA was synthesised from total RNA using the RevertAid First Strand cDNA Synthesis Kits (Fermentas, St. Leon-Rot, Germany) with VNdT_18 _primer.

### Identification of *HaGAS1 *and *HaCS*

For identification of sesquiterpene synthase genes by PCR, degenerate primers were used to obtain fragments of sunflower sesquiterpene synthases (forward primer, 5'-GAY GAR AAY GGI AAR TTY AAR GA-3', and reverse primer, 5'-CCR TAI GCR TCR AAI GTR TCR TC-3' [43]). PCR was performed in a total volume of 25 μl containing 1 μmol of the two primers, 0.25 μmol dNTPs, 1 unit of Taq DNA Polymerase (Fermentas, St. Leon-Roth, Germany) and 2 μl of cDNA. The PCR reaction was performed on a Mastercycler Gradient (Eppendorf, Hamburg, Germany) with 3 min of initial denaturation at 94°C, followed by 35 cycles of 1 min denaturation, 1 min annealing at 42°C, and 2 min of elongation at 72°C. Agarose gel electrophoresis showed a single band with a length of approximately 500 bp. Separation of the same PCR-reaction on 10% polyacrylamide gel revealed two bands with approximately 560 and 600 bp in length. Both bands were excised and transferred to 2 ml reaction tubes. After the addition of 150 μl ddH_2_O, the gel fragments were disrupted in a mixer mill for 1 min at 10 Hz using 2 ceramic beads. After centrifugation (5 min, 10,000 g), the supernatant was removed and used directly for reamplification of the PCR fragments with the same primer pair as before. The PCR products were gel-purified (Qiagen Gel Extraction Kit, Hilden, Germany) and used for direct sequencing using the same primers.

To obtain full length sequences, 3'-RACE was performed according to the protocol of Sambrook & Russell [65]. The reaction was carried out in a thermocycler with 5 min initial denaturation (94°C), 5 min of annealing at 49°C and a first elongation for 40 s at 72°C, followed by 30 cycles with 40 s denaturation (94°C), 1 min annealing (49°C), and 3 min elongation at 72°C. The PCR amplifications and subsequent direct sequencing of the resulting fragments were carried out using the RACE-adapter primer (5'-GAC TCG AGT CGG ACA TCG A-3' [65]) and the gene specific primer 5'-TTG AGA TTG AAA GGG AAA AC-3' for *HaGAS1 *and the gene specific primer 5'-CCA ACT AAG AAT AAG AGG AGA ATC-3' for *HaCS*. For identification of the 5'-ends of the mRNA sequences of *HaGAS1 *and *HaCS*, trichome total RNA was reversely transcribed to cDNA using VNdT_18 _primers. The enzyme assay was purified with the Eppendorf PCR Purification Kit (Hamburg, Germany) and a single stranded DNA-oligonucleotide (5'-ACT AGG ATC CAA GCT TGG AAT TCG TAC GTC TAG AGA TAT C-3', blocked by fluoresceine at the 3'-end, phosphorylated at the 5'-end) was ligated to the 3' end of cDNA by T4 RNA-ligase (Fermentas) at 37°C overnight [modified protocol from Edwards et al. and Troutt et al. [66,67]; T4-RNA ligase buffer (Fermentas), 20 μM ATP (Fermentas), 0.25 μg PEG 6000 (Roth GmbH, Karlsruhe, Germany) per μl ligation assay, 10 μg BSA (Fermentas) per μl ligation assay, 1 mM CoCl_2 _(Sigma-Aldrich, Taufkirchen, Germany), 25 nM DNA-oligonucleotide, 0.05 μl T4 RNA ligase per μl ligation assay]. The 5'-ends were amplified by PCR using gene-specific reverse primers (5'-GAC TTC AGA GTA ATA CGG CTC C-3' for *HaGAS1 *and 5'-GAC TTC AGA GTA ATA CGG CTC C-3' for *HaCS*) and a nested forward primer (5'-GAT ATC TCT AGA CGT ACG AAT C-3') for the ligated oligonucleotide at the 3' end of the cDNA. PCR was performed with 5 min initial denaturation, followed by 35 cycles with 40 s denaturation (94°C), 1 min annealing (52°C), 80 s elongation (72°C) and a final elongation step of 10 min using PCR reaction conditions as described above.

### Sequencing of the genomic DNA for *HaGAS1 *and *HaCS*

The genomic DNA (gDNA) sequences of *HaGAS1 *and *HaCS *were identified from genomic DNA isolated from *H. annuus *cv. HA300 leaves using the GenElute Plant Genomic DNA Miniprep Kit (Sigma-Aldrich GmbH, München, Germany). For subsequent amplification of the gDNA of *HaGAS1*, the following primer pairs were used: forward primer, 5'-CCT TCC ATC AAA TAA TTT TGA AG-3' and reverse primer, 5'-GTC TCT TGA AAC CTC ATA TCC-3'; forward primer, 5'-TGG TGC TAG ATG ACA CAT ATG AC-3' and reverse primer, 5'-CAC GAT TGA GAT ATT GTC CTA G-3'; forward primer, 5'-TTG AGA TTG AAA GGG AAA AC-3' and reverse primer, 5'-AGC ATC TTC ACT CAC TAT CTC AC-3'. The gDNA for *HaCS1 *was amplified with the following primer pairs: forward primer, 5'-TTG CAC CAA CTC CCA TTC-3' and reverse primer, 5'-GAC TTC AGA GTA ATA CGG CTC C-3'; forward primer, 5'-GGA GCC GTA TTA CTC TGA AGT C-3' and reverse primer, 5'-gga gcc gta tta ctc tga agt c-3'; forward primer, 5'-GAC TTC AGA GTA ATA CGG CTC C-3' and reverse primer, 3'-CCA ACT AAG AAT AAG AGG AGA ATC-5'. While amplification of *HaCS *gDNA revealed a single band, the PCR amplification of *HaGAS1 *resulted in two products with different length. The PCR products were gel-purified using the illustra GFX PCR Gel Band Purification Kit (GE Healthcare GmbH, Munich, Germany) and cloned into the pSC-A plasmid by UA-cloning following the instructions of the StrataClone PCR Cloning Kit (Stratagene Inc., La Jolla, USA). The fragments were sequenced. Sequences were aligned using the Seqman module of the DNASTAR software package (Lasergene, Madison, WI, USA). Introns and exons were identified by comparison of the gDNA sequences with the previously established mRNA sequences for *HaGAS1 *and *HaCS*. Both PCR products for *HaGAS1 *with the same primer pairs showed highly similar sequences in the exon parts but differed within the intron sequences in length and nucleotide composition. This resulted in the identification of a second germacrene A synthase gene (*HaGAS2*). For all sequencing work the sunflower cultivar HA300 was used, but the presence of all three sesquiterpene synthase genes was verified in wild type *H. annuus *(data not shown).

### Detection of the *HaGAS1 *and *HaGAS2 *transcripts in trichomes and roots

The full length coding sequence for *HaGAS1 *and *HaGAS2 *was amplified from trichome and root cDNA using the forward primer 5'-ATG GCA GCA AGT TGG AGC CAG-3' and the reverse primer 5'-TTA CAT GGG TGA AGA ACC AAC AAA C-3'. The PCR conditions were 2 min initial denaturation followed by 30 cycles of 40 s denaturation (94°C), 40 s annealing (60°C), 2 min 20 s elongation (72°C). The PCR amplicons were gel-purified. To distinguish between *HaGAS1 *and *HaGAS2 *amplicons, a restriction digestion was performed using *Pau*I and *Dra*I (Fermentas GmbH). The *HaGAS1 *contains a *Pau*I but no *Dra*I recognition site while *HaGAS2 *contains a *Dra*I site but no *Pau*I recognition site.

### Semi-quantitative RT-PCR and real-time quantitative RT-PCR

The cDNAs for RT-PCR were generated as described above. The constitutively expressed ubiquitin mRNA [68] served as the reference transcript. After reverse transcription, each cDNA sample was diluted several fold and used for PCR with the forward primer 5'-CAA AAC CCT AAC CGG AAA GA-3' and the reverse primer 5'-ACG AAG ACG GAG GAC GAG-3' to amplify ubiquitin cDNA. Equal initial cDNA concentrations within different samples were defined by equal amplification intensity for the ubiquitin transcript. PCR-Cycle number for PCR reactions was chosen to be in the linear range. Transcipts for *HaGAS1 *and *HaGAS2 *were traced by amplification with the forward primer 5'-TTG AGA TTG AAA GGG GAA AAC-3' and the reverse primer 5'-TGC CAA CAG AGT ATC TAG GTT CA-3'. To determine the expression level of *HaCS*, the forward primer 5'-CCA ACT AAG AAT AAG AGG AGA ATC-3' and the reverse primer 5'-GAC TTC AGA GTA ATA CGG CTC C-3' were used. The transcript for farnesyl diphosphate synthase [*FDPS*; Genbank: AF071887] was amplified with the following primer pair: forward primer 5'-ACT GCT TGT ACG GCT TTG CTT G-3' and reverse primer 5'-TTT CTT GCA TCT GCC CTT GGT TG-3'. For all semi-quantitative RT-PCR-experiments PCR products were separated on 1% agarose gels, stained for 30 min in a water bath containing 1.5 μg ml^-1 ^ethidium bromide and exposed to UV light (312 nm) for documentation.

For quantitative real-time PCR 5× master mixes (LightCycler FastStart DNA Master^PLUS ^SYBR Green I, Roche Diagnostics GmbH, Mannheim, Germany) were used according to manufacturer's recommendations on a LightCylcer 1.5 instrument (Roche Diagnostics GmbH). Same primer combinations as for semi-quantitative RT-PCR were used. Initial denaturation time for all samples was 10 min (95°C), followed by 50 cycles with 10s annealing (57°C for FPPS and HaCS, 59°C for HaGAS, 60°C for Ubiquitin), 20s elongation (72°C). Ramp temperature was set to 20°C/s. The melting curve of all samples was analyzed. Additionally, all reactions were loaded on 1% agarose gels to ensure amplification of products with the expected length. Relative gene expression was calculated using the Pfaffl method [69] with *Ubiquitin *as the reference transcript and compared to the expression levels of the specific transcripts of cDNA obtained from RNA isolations of glandular trichomes in presecretory stage.

### Heterologous expression of *HaGAS1*, *HaGAS2*, and *HaCS *in *E. coli*

The production of soluble proteins was made possible by expressing the proteins as N-terminal thioredoxin fusion proteins with the pET-32 EK/LIC plasmid (Novagen, Darmstadt, Germany). For generation of recombinant protein, the coding sequences for *HaGAS1 *and *HaGAS2 *were amplified with the forward primer 5'-GAC GAC GAC AAG ATG GCA GCA ATT GGA GC-3' and the reverse primer 5'-GAG GAG AAG CCC GGT TTA CAT GGG TGA AGA ACC AAC-3' from cDNA with KOD Polymerase (Novagen). The cDNA for *HaCS *was amplified using the forward primer 5'-GAC GAC GAC AAG ATG GCA ACA ACT GAA GC-3' and the reverse primer 5'-GAG GAG AAG CCC GGT TAC ATG GGG ACT GGA AC-3' and use of native Pfu-Polymerase (Fermentas). The PCR products were inserted in the pET-32 EK/LIC plasmid (Novagen, Darmstadt, Germany) according to the recommended protocol. The constructs were designated as pET32::*HaGAS1*, pET32::*HaGAS2*, and pET32::*HaCS*. These were used to transform NovaBlue cells (Novagen) which were grown overnight on Luria-Bertani (LB) plates supplemented with ampicillin (100 μg/ml). Plasmids were isolated from overnight cultures (LB medium supplemented with ampicillin) and their sequences were verified. Expression vectors for *HaGAS1 *and *HaGAS2 *were subcloned into the *E. coli *strain Rosetta-gami B (DE3)pLysS (Novagen), pET32::*HaCS *was subcloned into Rosetta 2 (DE3)pLysS cells (Novagen). Rosetta 2 cells were selected on LB-plates supplemented with carbenicillin (100 μg/ml), chloramphenicol (34 μg/ml). For Rosetta-gami B LB-plates kanamycin (15 μg/ml) and tetracycline (12.5 μg/ml) was also added.

### Heterologous expression of sesquiterpene synthase genes in *E. coli *and recombinant protein purification

LB-medium supplemented with carbenicillin (100 μg/ml) and chloramphenicol (34 μg/ml) was inoculated with 250 μl of an overnight culture of *E. coli *containing either pET32::*HaGAS1*, pET32::*HaGAS2*, or pET32::*HaCS *and grown at 37°C to an OD of 0.5 – 0.7 (600 nm). Cultures expressing *HaGAS1 *or *HaGAS2 *were shifted to 30°C over 30 min, induced with 0.5 mM isopropyl-β-d-thiogalactopyranoside (IPTG) and incubated for 4 h at 30°C (220 rpm). Cultures expressing *HaCS *were treated in the same way, but were shifted to 10°C over 45 min, induced with 0.1 mM IPTG and cultivated for a further 24 h at 10°C (180 rpm).

*E. coli *cells were concentrated by centrifugation (9,000 *g*, 5 min, 4°C) and proteins were extracted using 5 ml BugBuster Protein Extraction Reagent (Novagen) per g bacteria cells. 1 μl Benzoase (Novagen) per ml was added and the assay was incubated at room temperature (RT) for 20 min in a shaker (100 rpm). Insoluble proteins and cell fragments were removed by centrifugation (9,000 g, 25 min, 4°C) and the supernatant was cleared using a 0.45 μm filter. To every 5.0 ml protein extract 1.0 ml pre-equilibrated Ni-NTA agarose (Novagen) was added and incubated at 4°C for 60 min in a shaker. The complete slurry was transferred to chromatography column and the supernatant with the unbound proteins was removed. The agarose was washed twice with ice-cold wash buffer (50 mM NaH_2_PO_4_, 300 mM NaCl, 20 mM imidazol, pH 8.0), followed by a single washing step with 100 mM imidazol (same buffer as before). For elution of the 6x-His Tag proteins ice-cold 250 mM imidazol was used (same buffer as before). Soluble proteins were analyzed by SDS-PAGE.

Proteins were concentrated by ultracentrifugation (21,000 *g*, 20 min, 4°C) using Vivaspin 500 columns (exclusion size 30 kDa, Sartorius AG, Göttingen, Germany). The concentrated samples were desalted and diluted twice with enzyme assay buffer (ESB; 15 mM MOPSO, 10% glycerol, 1 mM ascorbic acid, 10 mM MgCl_2_, 1 mM MnCl_2_, pH 7.0; modified from Bennett et al. [32] and Bertea et al. [33]). After each step, the concentrated samples were diluted with ESB.

Functional characterization was performed in 2 ml reaction tubes. Between 200 and 400 μg recombinant protein dissolved in ESB was used within a reaction volume of 750 μl. Farnesyl diphosphate (FDP, Sigma-Aldrich GmbH, München, Germany) was added in a final concentration of 50 μM. The reaction assay was carefully overlaid with 250 μl pentane and incubated for 60 min at 30°C in a shaker (100 rpm). Afterwards, the assay was extracted by vigorous shaking, the pentane layer was removed and the reaction assay was extracted with another 250 μl pentane followed by extraction with 500 μl pentane/diethyl ether (1:4, v/v). The pentane extracts were combined and dried over a short column of aluminium oxide overlaid with MgSO_4 _in a Pasteur pipet. The pentane/diethyl ether extract was also passed over the aluminium oxide column. The column was washed with 1.5 ml diethyl ether. All extracts were combined and carefully concentrated to 50 μl under a constant nitrogen flow. For GC-FID measurements, 1 μl of the concentrated sample was directly injected. For GC-MS measurements, 25 μl of the sample were diluted with 375 μl pentane and 1 μl was injected.

### Enzyme characterization

For determination of catalytic properties, the recombinant fusion-proteins were digested with enterokinase to obtain the native protein. Digest was performed for 16 h at 20°C. Subsequently enterokinase was removed following the recommendations of the Enterokinase Cleavage Capture Kit (Novagen). To remove the cleaved thioredoxin fusion-part, affinity-chromatography on Ni-NTA agarose was performed. The flow-through, containing the native enzyme, was concentrated and diluted in ESB as described above and the proteins were analysed by SDS-PAGE. Protein concentrations were determined by the Bradford method [70]. Uncleaved *thioredoxin-HaGAS1 *fusion-protein was also used for determination of biochemical characteristics for comparison with the native *HaGAS1 *protein. Appropriate enzyme concentrations and incubation times were determined so that the reaction velocity was linear during the incubation time using 5 μM FPP (Sigma-Aldrich) spiked with [1-^3^H]FPP (Perkin Elmer, Rodgau-Jügesheim, Germany, 26.2 Ci/mmol).

A standard assay for determining biochemical properties was carried out in a final volume of 50 μl with 0.05 to 0.2 μg purified protein. The reactions were carefully overlay with 900 μl of dodecane and incubated for 15 min at 30°C in a thermoshaker-incubator (Thriller, Peqlab GmbH, Erlangen) at 300 rpm. Reactions were stopped by the addition of 50 μl of a solution containing 4 M NaOH and 1 M EDTA. To extract sesquiterpenes, the assays were vortexed for 1 min, centrifuged and 500 μl of the dodecane overlay was removed and mixed with 9.5 ml of liquid scintillation cocktail (Ultima Gold F, Perkin Elmer). Total radioactivity of the reaction products was determined using liquid scintillation counting (Wallac 1411 Liquid Scintillation Counter, Perkin-Elmer).

For pH optimum evaluation, assays were carried out using MES (pH 5.5, 6.0) MOPSO (pH 6.5, 7.0, 7.5) or Bis-tris propane (pH 8.0 to 9.5). These assays were done in duplicate. For determination of enzyme kinetics the concentration of FPP was varied from 0.125 to 30 μM with a fixed ratio of [1-^3^H]FPP. Ten different concentrations of FPP were used for each enzyme. Each concentration was done in triplicate. Calculation of the apparent *K*m value were obtained by Lineweaver-Burk plot analysis using Enzyme Kinetics!Pro software (ChemSW, Fairfield, USA).

### Protein expression *in vivo *in *S. cerevisiae*

For *in vivo *expression of the sesquiterpene synthases, the genes were cloned into the pESC-Leu2d plasmid [52]. *HaGAS1 *was amplified by PCR with the forward primer 5'-ACG TGC GGC CGC GAA CAT GGC AGC AGT TGG AGC CAG TG-3' and the reverse primer 5'-ACG TAG ATC TTT ACA TGG GTG AAG AAC CAA CAA ACA A-3'. *HaGAS2 *was amplified using the primer pair: forward primer 5'-ACG TCT CGA GAA TGG CAG CAG TTG GAG CCA GTG-3' and reverse primer 5'-ACG TGC TAG CTT ACA TGG GTG AAG AAC CAA CAA ACA A-3'. To generate the insert for *HaCS*, a PCR amplicon was generated using the forward primer 5'-ACG TCT CGA GAA TGG CAA CAA CTG AAG CTA ACA-3' and the reverse primer 5'-ACG TGC TAG CTT ACA TGG GGA CTG GAA CAC A-3'. The pET32::*HaGAS1 *and pET::*HaCS *plasmids served as template for the generation of the amplicons for *HaGAS1 *and *HaCS*. *HaGAS2 *was amplified from cDNA. To generate the thioredoxin fusion construct for *HaCS *in a yeast expression vector, the pET32 plasmid containing *HaCS *as a thioredoxin fusion-protein was amplified with the forward primer 5'-ACG TGG ATC CAA CAT GAG CGA TAA AAT TAT TCA C-3' and the reverse primer 5'-ACG TGC TAG CTT ACA TGG GGA CTG GAA CAC A-3'. As no germacrene A standard was available, the previously characterised germacrene A synthase (*LsLTC2*) from *Lactuca sativa *[32] was amplified from lettuce cDNA using the primer pair: forward primer 5'-ACG TGG ATC CAA CAT GGC AGC AGT TGA CAC TAA TG-3' and reverse primer 5'-ACG TGC TAG CTT ACA TGG ATA CAG AAC CAA C-3'. PCR reactions were performed using Phusion DNA Polymerase (New England Biolabs). *HaGAS2*, *HaCS*, and *LsLTC2 *were cloned into MCS2 of the pESC-Leu2d plasmid, *HaGAS1 *was cloned into MCS1 of the pESC-Leu2d plasmid. All amplicons were digested with the appropriate restriction enzymes overnight at 37°C, gel purified, and ligated into the pESC-Leu2d plasmid.

For all *in vivo *expression experiments, EPY300 *S. cerevisiae *cells were used. These cells were engineered for a high level of FDP production [51,52]. EPY300 were transformed with purified plasmids following the protocol from Gietz & Woods [71]. For protein expression, 5 ml SC medium (without Met, His, Leu and with 2% glucose supplement) were inoculated with single colony and grown overnight at 30°C (200 rpm). 250 ml culture flasks containing 50 ml YPAD medium (0.2% glucose, 1.8% galactose) supplemented with 1 mM methionine were inoculated with 1 ml overnight culture and overlaid with 5 ml of dodecane (Sigma-Aldrich GmbH, München, Germany). After 3–4 days incubation at 30°C (200 rpm), the cultures were transferred to 50 ml falcon tubes and centrifuged at 10,000 g (5 min). The dodecane overlay was carefully removed, diluted 1:100 with ethyl acetate and used directly for GC-FID and GC-MS analyses.

### Identification of products of enzyme expression *in vivo *in *S. cerevisiae *and of *in vitro *assays

GC-MS analyses of terpenes produced by recombinant enzyme in *S. cerevisiae *were performed on an Agilent 6890N gas chromatography system coupled to an Agilent 5975B mass spectrometer. *In vitro *assays with recombinant HaGAS1 and HaGAS2 protein, produced by heterologous expression in *E. coli*, were analyzed on an Agilent 6890N gas chromatograph coupled to an MS5973 mass spectrometer (Agilent). 1 μl samples were injected at 250°C and analysed on a HP-5MS column (30 m × 250 μm i.d. × 0.25 μm film thickness, Agilent). Helium (constant flow rate of 1 ml min^-1^) was used as carrier gas. The temperature program was 40°C for 1 min followed by a linear gradient of 10°C min^-1 ^to 250°C. Terpenes produced by *in vitro *assays with heterologously expressed *HaCS *were analyzed on a GC3400 gas chromatograph (Varian GmbH, Darmstadt, Germany) coupled to a Saturn 4D ion-trap mass spectrometer (Varian). 1 μl injection at a temperature of 250°C, He as carrier gas with 1.0 ml min^-1^. Temperature program: 50°C for 2 min, then 10°C min^-1 ^to 300°C, which was held for 3 min. Spectra were interpreted with NIST02 Mass Spectra Library (Wiley & Sons, Mississauga, Canada). Reference compounds were obtained from Sigma-Aldrich (δ-cadinene) or Fluka (β-Caryophyllene, Buchs, Swiss). α-copaene, α-humulene and α-muurolene were identified by comparison with characterized compounds of *Aloysia sellowii *oil (generous gift from J. Degenhardt, Jena). Alkane standard (C8-C20, Fluka) was used to determine retention indices.

For analysis of sesquiterpenes present in glandular trichomes, approximately 10,000 glandular trichomes were extracted with pentane (1 ml) for 10 min. The pentane extract was carefully concentrated using nitrogen gas and analysed by GC-MS. Headspace trap experiments for detection of emitted terpenes were done using solid phase micro extraction (SPME). Two young flower heads were placed in an Erlenmeyer flask covered with aluminium foil. After one hour the SPME fibre was placed in the flask together with the flower heads for 60 minutes and trapped volatiles were analysed by GC-MS (data not shown).

## Authors' contributions

JG conceived and carried out the experiments and drafted the manuscript. GM isolated germacrene A synthase 2. DKR designed the experiments for the yeast expression system, carried out experiments and revised the manuscript. OS devised experiments and coordinated the project. All authors read and approved the final manuscript.
